# Silver Anchored Polyaniline@Molybdenum Disulfide Nanocomposite (Ag/Pani@MoS_2_) for Highly Efficient Ammonia and Methanol Sensing under Ambient Conditions: A Mechanistic Approach

**DOI:** 10.3390/nano13050828

**Published:** 2023-02-23

**Authors:** Bandar A. Al-Mur, Mohammad Omaish Ansari

**Affiliations:** 1 Department of Environmental Sciences, Faculty of Meteorology, Environment and Arid Land Agriculture, King Abdulaziz University, Jeddah 21589, Saudi Arabia; 2 Center of Nanotechnology, King Abdulaziz University, Jeddah 21589, Saudi Arabia

**Keywords:** sensing, conductivity, MoS_2_, surfactant, polyaniline

## Abstract

We report the synthesis of silver anchored and para toluene sulfonic acid (*p*TSA) doped polyaniline/molybdenum disulfide nanocomposite (*p*TSA/Ag-Pani@MoS_2_) for highly reproducible room temperature detection of ammonia and methanol. Pani@MoS_2_ was synthesized by in situ polymerization of aniline in the presence of MoS_2_ nanosheets. The chemical reduction of AgNO_3_ in the presence of Pani@MoS_2_ led to the anchoring of Ag to Pani@MoS_2_ and finally doping with *p*TSA produced highly conductive *p*TSA/Ag-Pani@MoS_2_. Morphological analysis showed Pani-coated MoS_2_ along with the observation of Ag spheres and tubes well anchored to the surface. Structural characterization by X-ray diffraction and X-ray photon spectroscopy showed peaks corresponding to Pani, MoS_2,_ and Ag. The DC electrical conductivity of annealed Pani was 11.2 and it increased to 14.4 in Pani@MoS_2_ and finally to 16.1 S/cm with the loading of Ag. The high conductivity of ternary *p*TSA/Ag-Pani@MoS_2_ is due to Pani and MoS_2_ π–π* interactions, conductive Ag, as well as the anionic dopant. The *p*TSA/Ag-Pani@MoS_2_ also showed better cyclic and isothermal electrical conductivity retention than Pani and Pani@MoS_2,_ owing to the higher conductivity and stability of its constituents. The ammonia and methanol sensing response of *p*TSA/Ag-Pani@MoS_2_ showed better sensitivity and reproducibility than Pani@MoS_2_ owing to the higher conductivity and surface area of the former. Finally, a sensing mechanism involving chemisorption/desorption and electrical compensation is proposed.

## 1. Introduction

Nanomaterials that undergo a change in physical or chemical characteristics, such as color, optical properties, electrical conductivity, etc., on exposure to various gases, can be effectively used to monitor different types of volatile organic compounds (VOCs), toxic gases, environmental humidity, etc. [1,2]. Time and conductivity indicators can reliability track the nature of emitted gases and their toxicity, which is a vital function of sensors. In food samples, especially fish and meat, the humidity and biogenic amines present can also be related to food freshness or spoilage [3]. For example, the concentration of amines has been reported to increase from 130 to 330 ppm with the aging of fish samples and thus levels of gases and amines are a direct indicator of food freshness [4]. Apart from these, the environmental pollution caused by the release of toxic gases, such as NO_x_, SO_x_, Cox, etc., from vehicle fuel combustion and gas leakage from industrial plants and laboratories, is not only a threat to human health but also to animals and plants [5]. Thus large-scale, portable sensing devices are much needed to selectively monitor VOCs and gas leakages, as well as to predict the freshness of food items.

Sensors for the detection of amines and alcohols have recently attracted attention due to the widespread usage of these gases in a variety of sectors. For example, ammonia is used in the petroleum industry for its anticorrosive properties, as a curing agent in the leather industry, in food production as a source of nitrogen, and in agriculture, where it is used as a fertilizer [6]. However, as a toxic gas, its detection and the quantification of exposure are important for the safeguarding of human health. According to the Occupational Safety and Health Administration of the United States, the acceptable exposure limit over eight working hours is 25 ppm [7]. Exposure to higher levels may cause breathing issues and eventually death. Similarly, methanol, the simplest aliphatic alcohol, is listed as class B poison. Its exposure in higher quantities affects the central nervous system and the symptoms may include difficult breathing, drunkenness, eye irritation, blurred vision, blindness, loss of consciousness, vertigo, fatigue, convulsions, and possibility to death depending on the level of exposure. Thus, monitoring ammonia and methanol at workplaces and in the home is an important issue of concern.

Conducting polymers, such as polyaniline (Pani), polypyrrole, polythiophene, and their derivatives have shown gas-sensing properties at ambient conditions. However, most of these gas sensors suffer from low sensitivity for detection as well as irreversibility due to electrical compensation [8]. To solve this problem, improving the conductivity of a polymer and lowering the electrical compensation phenomenon is necessary to achieve an efficient polymer (or its composites) for gas sensors. Jia et al. [9] demonstrated that Pani-coated CNT showed a positive response and reproducibility towards ammonia at room temperature, which is mainly attributed to the synergism between the two. Tang et al. [10] showed that the high sensing response of polypyrrole-reduced graphene oxide composite is due to reduced graphene oxide, which provides an efficient pathway for electron transfer, thereby accelerating sensor response and recovery. Similarly, de Souza et al. [11] showed that Pani composite with CuO has significantly greater electrical conductivity than pure Pani. Apart from carbon and metal-based materials, noble metals, such as gold and silver, have also been utilized in combination with polymers. Kiani et al. [12] showed that a polypyrrole/silver nanocomposite is an efficient room-temperature ammonia gas sensor. Similarly, Kumar et al. [13] showed that for 100 ppm ammonia, the sensitivity of the Pani–Au composite increased to 52%, as compared to a mere 7% value for pure Pani.

Among different conducting polymers, Pani is widely researched, owing to characteristics such as its low cost, easily controllable redox states, good shelf life, etc. [14]. Similarly, MoS_2_ has attracted considerable attention as a nanomaterial, owing to its graphene-like structure, and good thermal and chemical stability [15]. Kim et al. [16] showed that the conductivity of Pani can be increased by incorporating it with MoS_2_. Similarly, an Ag–Pani composite prepared by Reda et al. [17] showed much higher conductivity than pure Pani. Thus, it can be concluded that the combination of Pani, MoS_2,_ and silver can yield a highly efficient sensing material, owing to the high conductivity of all the constituents. Accordingly, in this work para toluene sulfonic acid (*p*TSA) doped silver decorated Pani@MoS_2_ nanocomposite was prepared by coating Pani onto hydrothermally prepared MoS_2_ and subsequently anchoring Ag to it. This prepared *p*TSA/Ag-Pani@MoS_2_ was studied for its morphological and structural characteristics. Finally, ternary Ag-Pani@MoS_2_ was studied for its electrical conductivity with regard to its application as an ammonia and methanol sensor.

## 2. Experimental Section

### 2.1. Material and Methods

Aniline monomer, oxidant-potassium persulfate (PPs), HCl (35%), AgNO_3_ (99.99%), MoO_3_, thiourea, ascorbic acid, ethanol, and NH_4_OH were obtained from Sigma Aldrich (St. Louis, MO, USA). Aniline monomer was distilled twice under reduced pressure before use. The *p*TSA and cetyl trimethyl ammonium bromide (CTAB) were obtained from Otto Chemicals.

The synthesized *p*TSA/Pani, *p*TSA/MoS_2_@Pani, and *p*TSA/Ag-MoS_2_@Pani were characterized for their morphological and topographical features by scanning electron microscopy (SEM; JSM7600F, JEOL, Tokyo, Japan) and transmission electron microscopy (JEOL ARM-200F, HRTEM, Tokyo, Japan).

The structural characterizations were performed by X-ray photoelectron spectroscopy (XPS; ESCALAB 250, Thermo Fisher Scientific, Warrington, UK, used at a monochromatized Al Kα X-ray source λ ¼ 1486.6 eV), X-ray diffraction (XRD; ALTIMA-IV, RIGAKU) and Raman spectroscopy (DXR 532 Raman Microscope, Thermo Scientific, Madison, WI, USA).

### 2.2. DC Electrical Conductivity Retention and Ammonia Sensing Studies

DC electrical conductivity was measured by a 4-in-line probe device connected to a temperature controller (PID-200). The calculations were performed by using the following equations:

*ρ = ρo/G7(W/S)* (1)

*G7 (W/S) = ln2 (2S/W)* (2)

*ρo = 2πS(V/I)* (3)

*σ = 1/ρ* (4)

Here, *G7(W/S)*—the correction factor—depends on sample thickness and probe spacing; *I* is current (A), *V* is the voltage (V), *W* is sample thickness (cm), and *S* is probe spacing (cm). The *ρ* is the resistivity, *ρo* is the specific resistivity (Ω-cm), and *σ* is the conductivity (S/cm) [18].

The thermal stability of the electrical conductivities of *p*TSA/Pani, *p*TSA/Pani@MoS_2,_ and *p*TSA/Ag-Pani@MoS_2_ was analyzed using cyclic and isothermal heating experiments and subsequent measurements of DC electrical conductivity. For isothermal studies, the pellet of the nanocomposite was heated to temperatures of 50, 70, 90, 110, and 130 °C for 40 min, and conductivity values were recorded at intervals of 10 min. In the cyclic studies, the nanocomposite pellet was heated from 40–150 °C five times at an interval of 1 h [19]. The details of the gas sensing set-up and studies can be seen elsewhere [20]. Prior to the cyclic, isothermal and gas sensing measurements the samples were annealed at 150 °C for 2 h.

### 2.3. Fabrication of pTSA/Ag-Pani@MoS_2_

For the synthesis of *p*TSA/Ag-Pani@MoS_2_, firstly, MoS_2_ nanosheets were prepared, then coated with Pani to create Pani@MoS_2,_ and finally, Ag was anchored onto Pani@MoS_2_ to produce *p*TSA/Ag-Pani@MoS_2_. For the synthesis of MoS_2_ nanosheets, 0.23 g of MoO_3_ and 0.53 g of thiourea were stirred in 70 mL of water for 1 h, and then the whole reaction mixture was charged into a 100 mL Teflon-lined hydrothermal reactor, and subsequently heated at 200 °C for 20 h. The resulting black MoS_2_ precipitate was separated by centrifugation, washed with solvents (water and ethanol), and subsequently dried at 80 °C for 12 h. The Pani@MoS_2_ was synthesized by in situ oxidative polymerization of aniline in the presence of MoS_2_. In a typical process, 0.5 g of MoS_2_ was vigorously stirred in 100 mL of 1M HCl solution with the addition of 0.25 g of CTAB and 5 mL of aniline. The whole reaction mixture was constantly stirred for 1 hr for the proper adsorption of aniline over MoS_2_. Subsequently, the oxidant solution (7.4 g of PPs in 100 mL 1M HCl) was added to the above mixture to initiate the polymerization process. The whole reaction mixture was constantly stirred for 24 h, followed by centrifugation to collect MoS_2_@Pani and subsequent washing with solvents (water and ethanol). Thus, prepared wet Pani@MoS_2_ was further doped with *p*TSA by stirring in *p*TSA solution (1 g *p*TSA in 100 mL water) for 30 min, followed by separation with centrifugation and subsequent drying at 80 °C for 12 h. For the Ag-MoS_2_@Pani, the wet MoS_2_@Pani was stirred in 100 mL water, with the subsequent addition of 0.30 g of ascorbic acid, followed by the addition of 0.5 g of AgNO_3_, leading to the precipitation of Ag over MoS_2_@Pani. The prepared Ag-Pani@MoS_2_ was washed with solvents and subsequently doped with *p*TSA as per the procedure described earlier.

## 3. Results and Discussion

**Figure 1:**
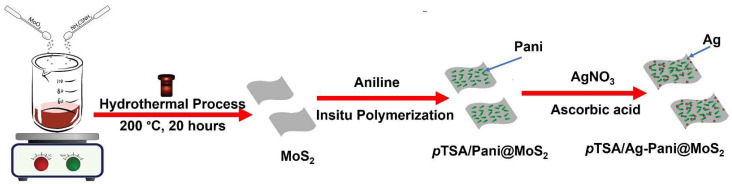
Schematic diagram representing the fabrication of *p*TSA/Ag-Pani@MoS_2_.

A hydrothermal methodology was employed for the MoS_2_ sheets and its composite with Pani was obtained by in situ oxidative polymerization of aniline in its presence. Finally, Ag nanoparticles were anchored onto the Pani@MoS_2_ by the reduction of AgNO_3_. This synthesized *p*TSA/Ag-Pani@MoS_2_ nanocomposite is expected to show a strong electrical response and gas sensing characteristics owing to the additional effect of the conductive Pani, MoS_2,_ and Ag. Figure 1 presents a schematic diagram of the synthesis of *p*TSA/Ag-Pani@MoS_2_ nanocomposites.

### 3.1. Morphological Analysis

**Figure 2:**
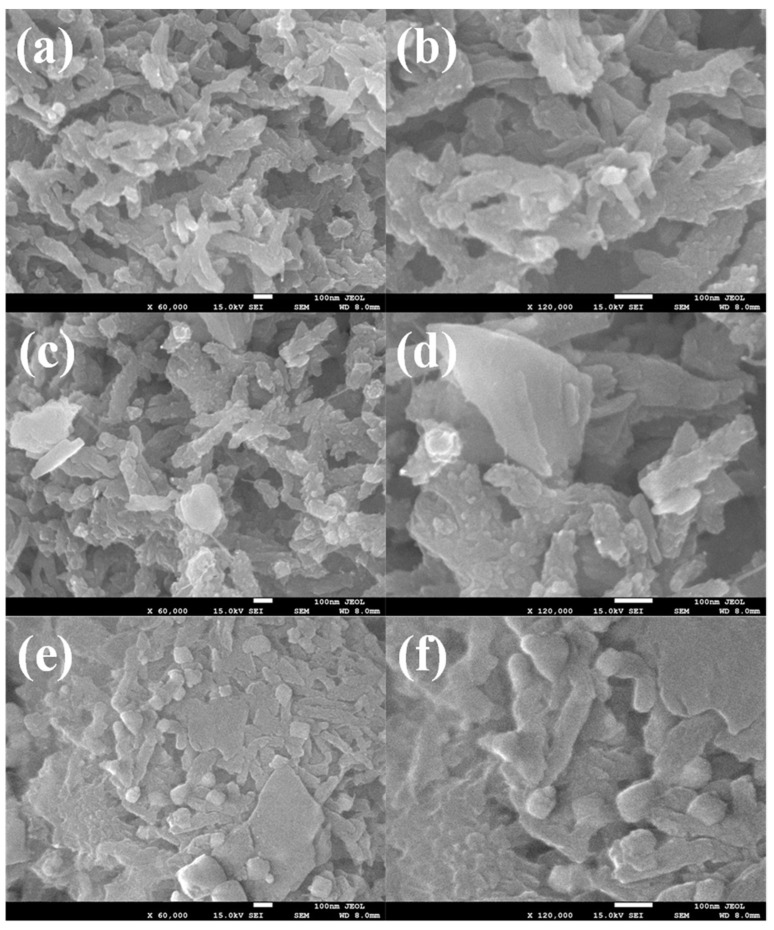
SEM images of *p*TSA/Pani (**a**,**b**), *p*TSA/Pani@MoS_2_ (**c**,**d**), and *p*TSA/Ag-Pani@MoS_2_ (**e**,**f**) at different magnifications.

The SEM analysis of *p*TSA/Pani, *p*TSA/Pani@MoS_2,_ and *p*TSA/Ag-Pani@MoS_2_ is presented in Figure 2. The *p*TSA/Pani shows mostly interconnected fibrous structures with the presence of other structures, such as stacked globules and sheets. As interpreted by the naked eye, the Pani fibers seem to be of two types: either smooth and thin, or thick and rough. It can be interpreted that during the rapid mixing technique, some fibers adsorbed more aniline or oxidant during their genesis and grew more along the radial axis, becoming thicker, or generating other structures. Apart from this, some porosity can also be seen between different agglomerates. In the case of *p*TSA/Pani@MoS_2_, large floating sheets of MoS_2_, Pani tubes, and Pani coating over MoS_2_ covering large areas, as well as small agglomerates of Pani, can be seen. In the case of ternary *p*TSA/Ag-Pani@MoS_2_, Pani-coated MoS_2_ is visible, along with Ag spheres and tubes well anchored to the surface. The SEM images of pure MoS_2_ sheets is presented in Figure S1.

**Figure 3:**
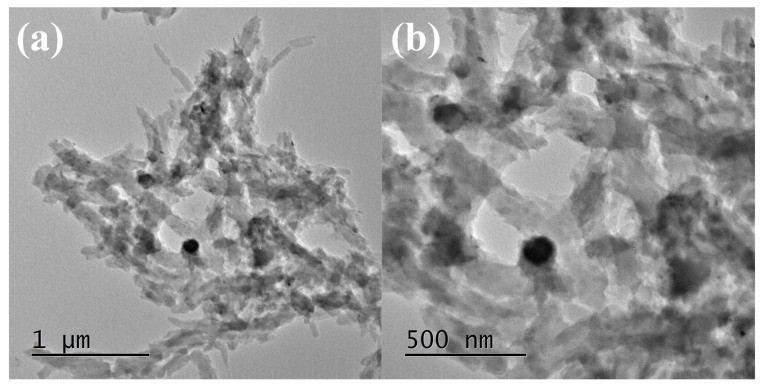
(**a**,**b**) TEM images of *p*TSA/Ag-Pani@MoS_2_ at different magnifications.

**Figure 4:**
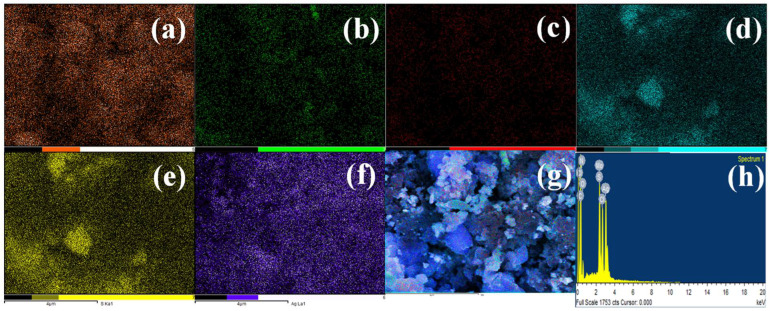
Mapping analysis of *p*TSA/Ag-Pani@MoS_2_: (**a**) C, (**b**) O, (**c**) N, (**d**) Mo, (**e**) S, (**f**) Ag, and (**g**) all elements mixed; (**h**) EDAX analysis of *p*TSA/Ag-Pani@MoS_2_.

The TEM analysis showed well dispersed Pani and Ag inside/on the MoS_2_ sheets (Figure 3). It can be clearly seen that all the constituents are well linked, which is anticipated to create an interconnected network for charge transfer. The dimensions of the MoS_2_ platform are in the range of micrometers and Pani tubes of diameter ~100 nm. Ag nanoparticles are in the range of 20–30 nm in diameter, except for a few larger particles in the 60–100 nm range. The EDAX analysis showed the presence of C, O, N, Mo, S, and Ag (Figure 4) while the elemental mapping showed their uniform mixing, thereby suggesting the efficacy of the synthesis methodology.

### 3.2. Structural Analysis

#### 3.2.1. XRD

**Figure 5:**
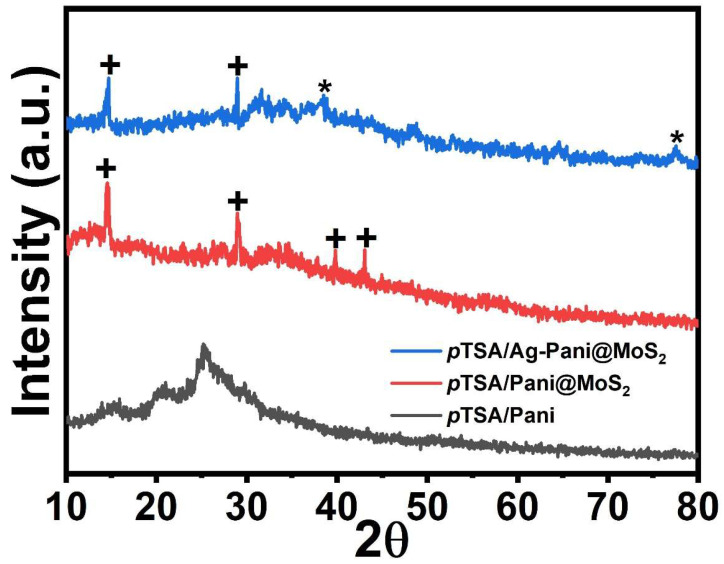
The XRD patterns of *p*TSA/Pani, *p*TSA/Pani@MoS_2,_ and *p*TSA/Ag-Pani@MoS_2_ nanocomposites. The (+) indicate peaks of MoS_2_ and (*) corresponds to the peak of Ag.

The XRD of *p*TSA/Pani, *p*TSA/Pani@MoS_2,_ and *p*TSA/Ag-Pani@MoS_2_ is presented in Figure 5. Pure Pani shows semi-crystalline features due to the presence of benzenoid and quinonoid groups with the observance of three diffraction peaks at 15.7, 20.7, and 25.2 2θ [21]. Padmapriya et al. [22] showed that the peaks at 15.7, 20.7, and 25.2 2θ correspond to (121), (113), and (322) crystal planes, thereby suggesting that most of the Pani is oriented along them. The *p*TSA/Pani@MoS_2_ showed peaks at 14.58, 29, 39.76, and 43 2θ, corresponding to MoS_2_ [23]. In the case of *p*TSA/Ag-Pani@MoS_2_, apart from MoS_2_ peaks, the peaks 38.13, and 77.67 2θ correspond to the (111) and (311) crystallographic planes of Ag nanoparticles with a cubic structure (JCPDS card No. 03-065-2871) [24]. The peaks of Pani are also not distinct, owing to the high-intensity peaks of MoS_2_ and Ag, which suppress the peaks of Pani along the Y axis of the graph due to the presented scale.

#### 3.2.2. XPS

**Figure 6:**
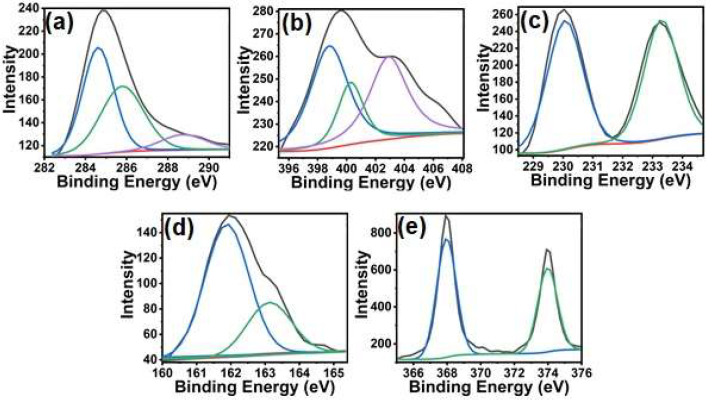
Deconvoluted XPS of *p*TSA/Ag-Pani@MoS_2_: C1s (**a**), N1s (**b**), Mo3d (**c**), S2p (**d**), and Ag3d (**e**).

The elemental details, functional groups, and their interactions were analyzed by XPS (Figure 6). The full-range survey scan showed the presence of C, O, N, Cl, and Ag without the observation of any other impurities (Figure S2). The carbon peak is due to the residual carbon from the sample and the instrument. The C1s peaks can be deconvoluted into three peaks at 284.61, 285.8, and 288.21 eV, corresponding to sp2 and sp3-hybridized carbon (C–C, C=C), C–N and C=O, respectively [25,26,27]. The N1s peak shows peaks at 399, 400.28, and 402.90 eV, corresponding to the quinoid imine (–N=), benzenoid amine (–NH−), and the positively charged polaron species (N+), respectively [28]. The peaks at 229.8 and 232.5 eV correspond to Mo^4+^ 3d5/2 and Mo^4+^ 3d3/2, while the peaks at 161.9 and 163.2 eV are assigned to S2–2p3/2 and S2–2p1/2 of MoS_2_, respectively [29]. The Ag peak can be deconvoluted into the peaks at 367.90 and 373.90 eV and is attributed to the metallic silver Ag (0), thus confirming its successful deposition on *p*TSA/Ag-Pani@MoS_2_ [30].

### 3.3. DC Electrical Conductivity

**Figure 7:**
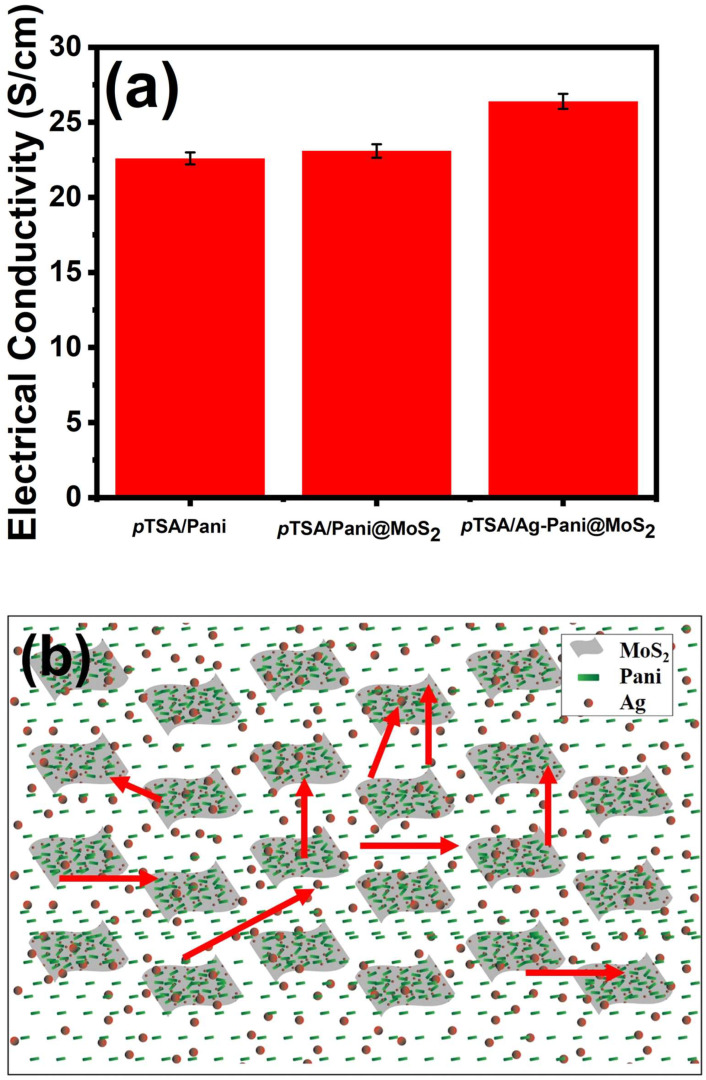
(**a**) Initial electrical conductivity of unannealed *p*TSA/Pani, *p*TSA/Pani@MoS_2_ and *p*TSA/Ag-Pani@MoS_2_ nanocomposite, and (**b**) conduction of charge carriers along Pani, Ag and MoS_2_.

The DC electrical conductivity of the *p*TSA/Pani, *p*TSA/Pani@MoS_2,_ and *p*TSA/Ag-Pani@MoS_2_ was measured by a 4-in-line probe as presented in Figure 7a. The doping acids were *p*TSA and HCl, as CTAB cannot act as a dopant due to its large cationic size [31]. The electrical conductivity of unannealed *p*TSA/Pani, *p*TSA/Pani@MoS_2_, and *p*TSA/Ag-Pani@MoS_2_ at room temperature is 22.6, 23.1, and 26.4 S/cm, respectively, and thus the composites show much higher conductivity than our previously reported mineral acid doped composites [19]. The annealed samples at 150 °C showed slightly lower electrical conductivities i.e., 11.2, 14.4 and 16.1 S/cm for *p*TSA/Pani, *p*TSA/Pani@MoS_2,_ and *p*TSA/Ag-Pani@MoS_2_ respectively which might be due to the loss of moisture and impurities. The reason for the higher electrical conductivity here in contrast to mineral acid doped Pani is due to the high charge density, owing to the presence of HCl and *p*TSA, decreased hopping or tunneling of charge carriers, and higher metallic regions in the composite [2]. The incorporation of MoS_2_ and Ag in Pani also leads to higher electrical conductivity in ternary *p*TSA/Ag-Pani@MoS_2_ due to the synergistic or additional effect of Pani, MoS_2,_ and Ag, since both MoS_2_ and Ag have been reported to increase electrical conductivity. Kim et al. [16] showed that Pani@MoS_2_ is more conductive than pure Pani, due to the increase in the compactness of Pani on the incorporation of MoS_2_. The aligned Pani fibers or coating over MoS_2_ results in an increase in the compactness of Pani in *p*TSA/Pani@MoS_2_ and *p*TSA/Ag-Pani@MoS_2_. Another reason is the π–π* interactions between Pani and MoS_2_ lead to the greater mobility of charge carriers in *p*TSA/Pani@MoS_2_ and *p*TSA/Ag-Pani@MoS_2_. Similarly, Xia et al. [32] reported that Ag can successfully enhance the electrical conductivity of Pani and thus the presence of both MoS_2_ and Ag will significantly increase its conductivity, as obtained in our analyses. Another reason is that the in situ polymerization of aniline over MoS_2_ provides much greater surface areas of Pani, leading to a much extended and conjugated system, which subsequently produces a much greater charge carrier density. Figure 7b represents the movement of charge carriers (through Pani, through Ag, π–π* interactions between Pani and MoS_2_, through Pani and Ag, etc.).

**Figure 8:**
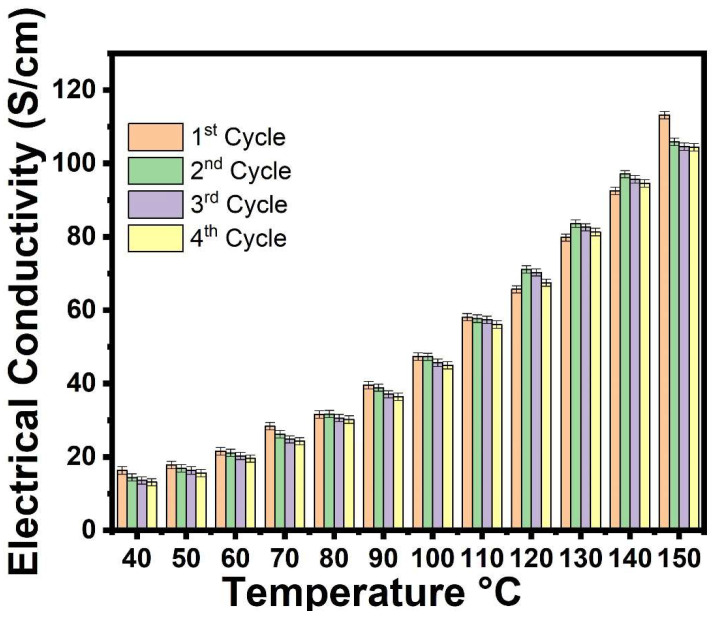
DC electrical conductivity retention of *p*TSA/Ag-Pani@MoS_2_ nanocomposite under cyclic aging conditions.

The testing of DC electrical conductivity of *p*TSA/Ag-Pani@MoS_2_ through the cyclic aging technique showed an increase in conductivity at higher temperatures in all cycles (Figure 8). Conductivity decreased in all cycles, however, from the second cycle onwards, when the rise in conductivity at higher temperature was much more uniform. The reason for the loss of conductivity is the loss of dopant acids (HCl and *p*TSA), moisture, and other volatile components. However, it is expected that considerable ordered chain alignment occurs at higher temperatures, leading to uniformity in electrical properties. The increase in electrical conductivity with increasing temperature is due to normal thermal activation, as the mobility of charged carriers increases via the conductive chains of Pani, the π–π* interactions between Pani and MoS_2,_ and through conductive Ag. The loss of electrical conductivity decreased in subsequent cycles, with the third and fourth cycles showing much smaller losses (0.77 to 0.53 S/cm, respectively), thereby suggesting an increase in semiconducting properties on annealing at higher temperatures.

**Figure 9:**
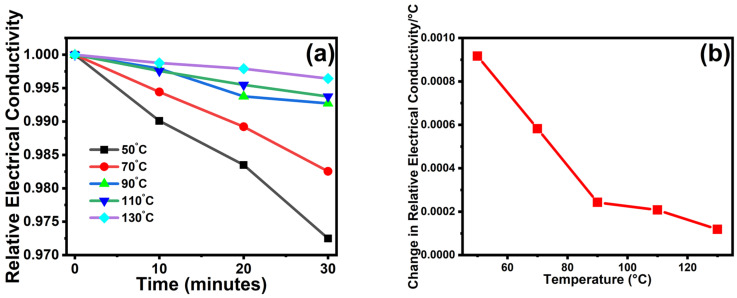
(**a**,**b**) DC electrical conductivity retention of *p*TSA/Ag-Pani@MoS_2_ nanocomposite under cyclic aging conditions at 50, 70, 90, 110, and 130 °C.

The DC electrical conductivity retention of annealed *p*TSA/Ag-Pani@MoS_2_ was plotted as the change in the DC electrical conductivity divided by the duration of the experiment (30 min) [18]. The relative loss in conductivity of *p*TSA/Ag-Pani@MoS_2_ decreased with temperature, but the loss was very much similar (in the range of 17 × 10^−3^ to 59 × 10^−3^), thereby confirming the semiconducting nature of *p*TSA/Ag-Pani@MoS_2_ under aging conditions (Figure 9).

The DC retention studies showed much higher conductivity and stability of *p*TSA/Ag-Pani@MoS_2_ at higher temperatures when compared with Pani composites doped with mineral acids (HCl, H_2_SO_4_, HNO_3_, etc.). Here the *p*TSA/Ag-Pani@MoS_2_ showed no loss in conductivity even at 150 °C, in comparison to previous reports that show a loss in conductivity around 120 °C. Given the minimal loss at higher temperatures, it can be interpreted that *p*TSA has a major effect on conductivity, leading to a nanocomposite with high electrical stability.

### 3.4. Vapor Sensing Studies

**Figure 10:**
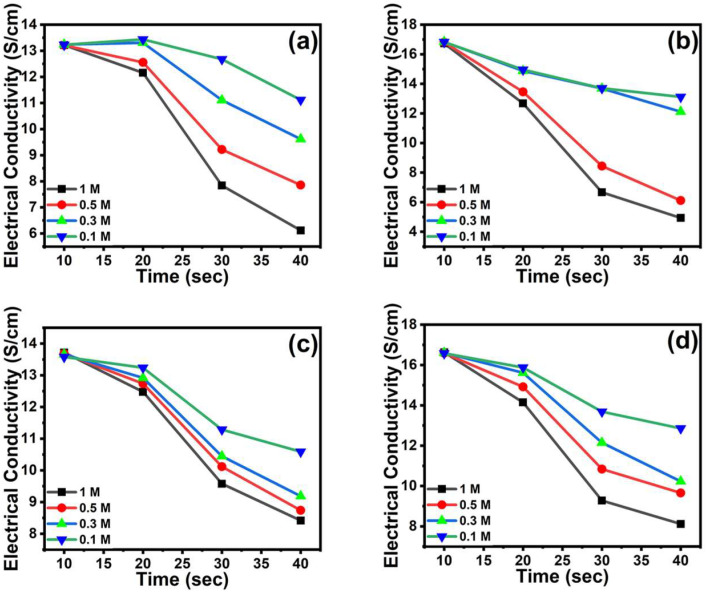
Exposure of *p*TSA/Pani@MoS_2_ to ammonia (**a**) and methanol (**c**); exposure of *p*TSA/Ag-Pani@MoS_2_ to ammonia (**b**) and methanol (**d**).

The vapor sensing of *p*TSA/Ag-Pani@MoS_2_ for ammonia and methanol was studied in the range of 0.1–1 M for 40 s (Figure 10). A sharp decrease in the conductivity was observed at high concentration but the sensitivity decreased with the decrease in concentration. This response results suggests that a concentration of ammonia and methanol even lower than 0.1 M can be efficiently sensed by *p*TSA/Ag-Pani@MoS_2_. The percentage sensing response (SR) was calculated by [33]:

SR (%) = (Change in electrical conductivity/Initial conductivity) × 100(5)

The percentage sensing response of *p*TSA/Ag-Pani@MoS_2_ and *p*TSA/Pani@MoS_2_ for 1 M ammonia was 70.48 and 53.70% and for methanol, 51.17 and 38.62%, respectively. This suggests that both *p*TSA/Ag-Pani@MoS_2_ and *p*TSA/Pani@MoS_2_ are more responsive towards ammonia, with *p*TSA/Ag-Pani@MoS_2_ showing much higher sensitivity towards both ammonia and methanol.

**Figure 11:**
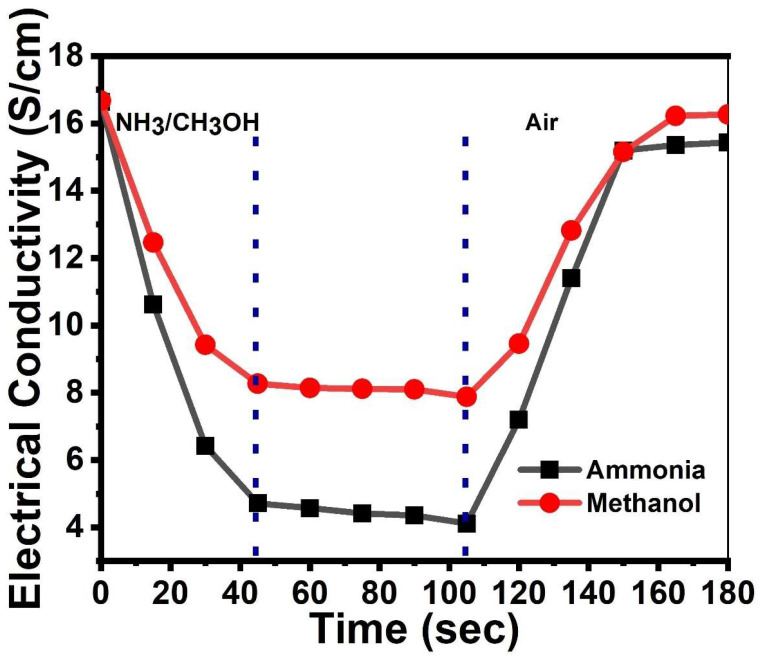
Limit of DC electrical conductivity saturation of *p*TSA/Ag-Pani@MoS_2_ on exposure to ammonia and methanol.

On account of the higher sensitivity of *p*TSA/Ag-Pani@MoS_2_ in relation to both ammonia and methanol, its stability was tested for long-term exposure to vapors. Figure 11 shows that on exposure to ammonia and methanol, *p*TSA/Ag-Pani@MoS_2_ attained a steady state in ~45 s, with conductivity in the case of ammonia decreasing from 16.64 to 4.12 S/cm and for methanol from 16.68 to 7.88 S/cm. On further exposure over a subsequent 60 s, very little drop in conductivity was observed, as indicated by a nearly horizontal line in the graph. Furthermore, on flushing the sample with air, the conductivity acquired close to its original value in both ammonia- and methanol-exposed samples. However, it can be observed that in the case of ammonia- and methanol-exposed samples, a 7.3 and 2.5% loss in conductivity was observed, respectively.

**Figure 12:**
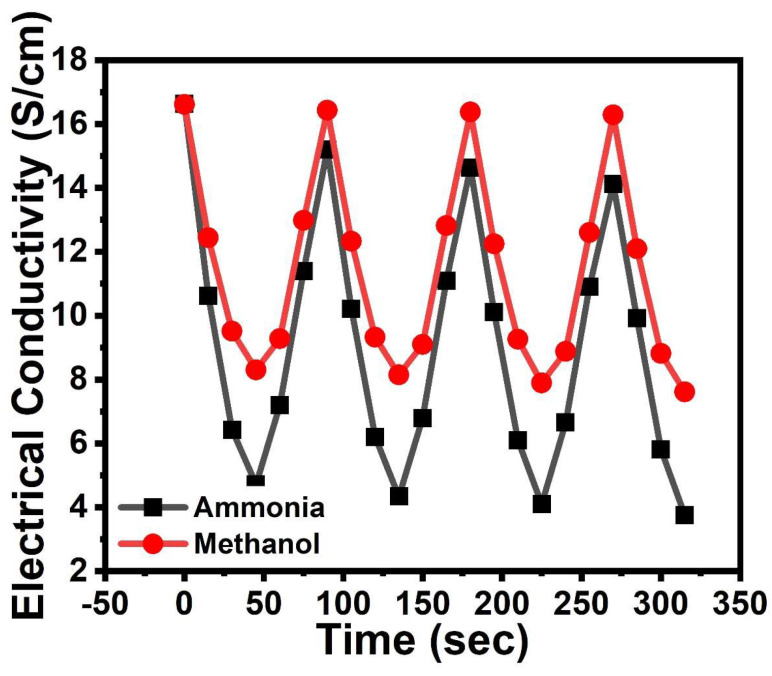
Reversibility studies of *p*TSA/Ag-Pani@MoS_2_ on ammonia and methanol exposure.

For the reproducibility studies, the *p*TSA/Ag-Pani@MoS_2_ sample was exposed to vapors for 45 s, followed by exposure to air, and conductivity was recorded [34] (Figure 12). It can be seen from the figure that *p*TSA/Ag-Pani@MoS_2_ possesses high reversibility in relation to both ammonia and methanol. The conductivity changes of 5.24 and 3.63 S/cm for ammonia and methanol, respectively, after 45 s suggests the higher sensitivity of *p*TSA/Ag-Pani@MoS_2_ towards ammonia. However, a loss of sensitivity of 1.44 and 0.18 S/cm, respectively, in the ammonia and methanol-exposed samples during the first cycle suggests much greater electrical compensation in the case of ammonia. It can also be seen that the loss of ~15 and ~2% conductivity was observed for ammonia- and methanol-exposed samples after three cycles. From the observations, it can be concluded that although *p*TSA/Ag-Pani@MoS_2_ showed much higher sensitivity towards ammonia, it can also be used in longer runs for methanol sensing.

### 3.5. Selectivity

**Figure 13:**
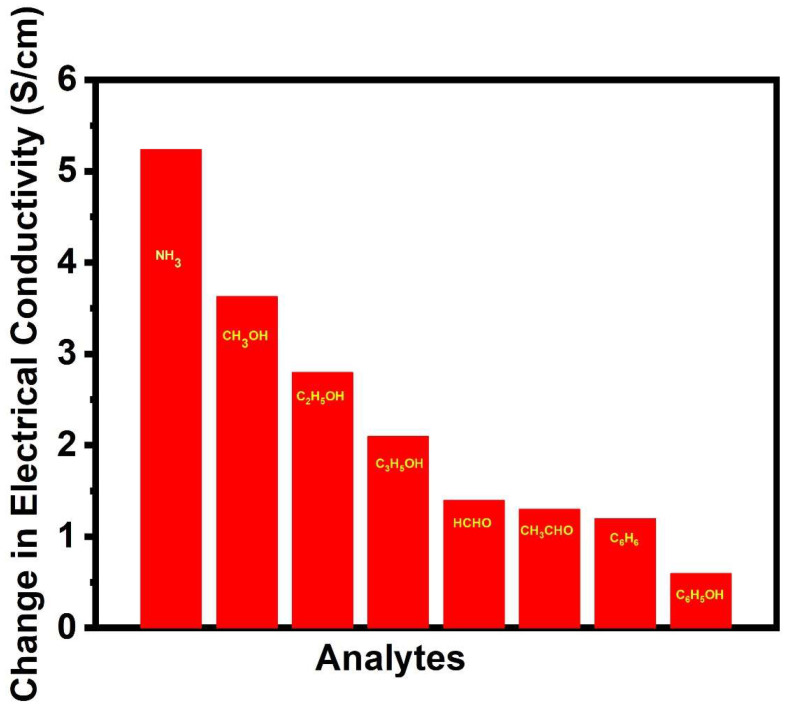
Change in the electrical conductivity of *p*TSA/Ag-Pani@MoS_2_ on exposure to different analytes.

For the selectivity studies, *p*TSA/Ag-Pani@MoS_2_ was exposed to different analytes under similar conditions (1M of 30 mL of analytes). The sensitivity towards ammonia was the highest, followed by alcohols, aldehydes, benzene, and phenol (Figure 13). It can be interpreted that the change in conductivity depends on various factors, such as electron donation capacity, vapor pressure, size of the analyte, as well as electrical compensation capacity towards *p*TSA/Ag-Pani@MoS_2_. Both ammonia (owing to its high basicity and compensation effect) and methanol (among other alcohols) interact much more efficiently with imine nitrogen than ethanol and propanol, due to their much smaller size. Benzene and phenol showed the least change in conductivity, due to their much lower vapor pressure and larger size, which resulted in the least interaction between charged nitrogen and Pani.

### 3.6. Sensing Mechanism

The sensing behavior of Pani is recorded as the change in its electrical conductivity. The interaction of vapors with positively charged nitrogen hinders the movement of charge carriers (solitons, polarons, or bipolarons), which results in an increase/decrease in electrical conductivity. This is the main principle employed in the fabrication of Pani-based gas sensors.

**Figure 14:**
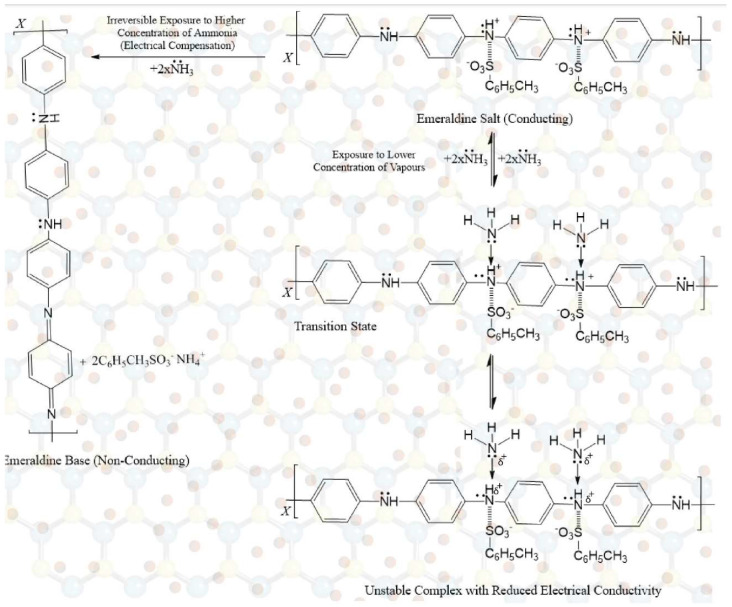
The mechanism for ammonia sensing by *p*TSA/Ag-Pani@MoS_2_.

**Figure 15:**
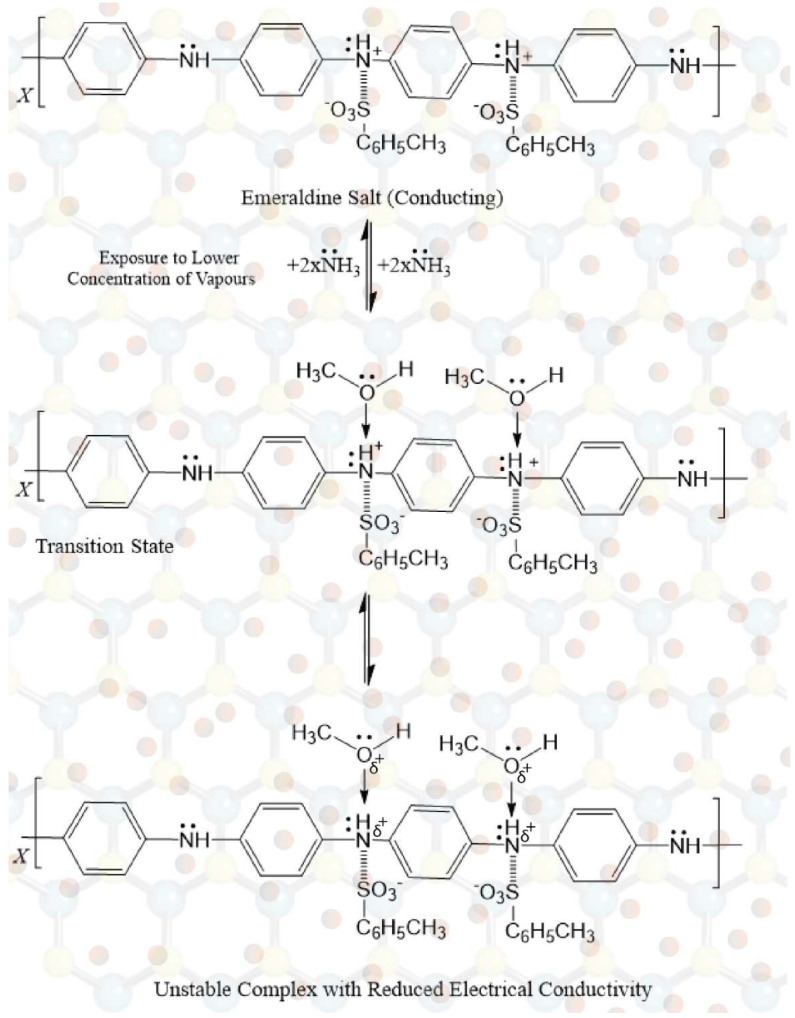
The mechanism for methanol sensing by *p*TSA/Ag-Pani@MoS_2_.

The sensing mechanism involves the chemisorption–desorption of ammonia/methanol onto *p*TSA/Ag-Pani@MoS_2_ and the electrical compensation phenomenon. On exposure to lower concentrations of vapors, the lone pair of ammonia/methanol interacts with the positive nitrogen in Pani, which results in the lowered mobility of charge carriers in Pani and hence a decrease in conductivity. However, as soon as the chemisorption of ammonia/ethanol occurs, the valency of nitrogen and oxygen increases, and an unstable configuration is obtained, as shown in Figure 14 and Figure 15. Thus, possible desorption occurs, leading to the restoration of electrical conductivity. On exposure to higher concentrations of ammonia, the acid-base reaction between *p*TSA and ammonia predominates, leading to the electrical neutralization of Pani into an emeraldine base and hence a loss of sensing properties. In contrast, due to little or no compensation by methanol, it can be suggested that *p*TSA/Ag-Pani@MoS_2_ composite can be used for the sensing of methanol for a longer number of cycles as well as at higher concentrations. The improved sensing performance of *p*TSA/Ag-Pani@MoS_2_, when compared with *p*TSA/Pani@MoS_2_, is due to the greater surface area and more conductive pathways in the composite. The surface area of *p*TSA/Pani@MoS_2_ and *p*TSA/Ag-Pani@MoS_2_ is presented in Figure S3 and Table S1.

## 4. Conclusions

In summary, *p*TSA/Ag-Pani@MoS_2_ was synthesized by in situ polymerization of aniline with hydrothermally synthesized MoS_2_ and the subsequent deposition of silver nanoparticles over it. Morphological studies observed Pani-coated MoS_2_ Ag spheres and tubes well anchored to the surface. Structural analysis showed the presence and interactions between Pani, MoS_2_, and Ag, thereby suggesting the successful synthesis of the ternary composite. The ternary composite showed the highest electrical conductivity of 16.1 S/cm, due to the Pani and Ag offering multiple conduction pathways, i.e., through Pani, through Ag, π–π* interactions between Pani and MoS_2_, through Pani and Ag, etc. Cyclic aging studies demonstrated a much lower loss of conductivity during the third and fourth cycles, i.e., 0.77 to 0.53 S/cm, respectively, while isothermal measurement showed a similar loss at (in the range of 17 × 10^−3^ to 59 × 10^−3^), thereby suggesting the semiconducting nature of the composite. The *p*TSA/Ag-Pani@MoS_2_ showed better sensing responses of 70.48 and 51.17%, respectively, for ammonia and methanol, in contrast to 53.70 and 38.62% for that of *p*TSA/Pani@MoS_2_. The sensing mechanism showed that chemisorption–desorption of ammonia/methanol onto *p*TSA/Ag-Pani@MoS_2_ occurs, while the compensation phenomenon also takes place in the case of ammonia.

## Data Availability

The data presented in this study are available on request from the corresponding author.

## Supplementary Materials

The following supporting information can be downloaded at: https://www.mdpi.com/article/10.3390/nano13050828/s1, Figure S1: SEM of MoS_2_ nanosheets; Figure S2: XPS survey scan of *p*TSA/Ag-Pani@MoS_2_; Figure S3: Absorption/desorption isotherm plots of *p*TSA/Pani@MoS_2_ and *p*TSA/Ag-Pani@MoS_2_; Table S1: Surface area and pore size profile of *p*TSA/Pani@MoS_2_ and *p*TSA/Ag-Pani@MoS_2_.
