# Supplementary material for: Silver Anchored Polyaniline@Molybdenum Disulfide Nanocomposite (Ag/Pani@MoS2) for Highly Efficient Ammonia and Methanol Sensing under Ambient Conditions: A Mechanistic Approach
Source: Nanomaterials (Basel). 2023 Feb 23;13(5):828. doi: 10.3390/nano13050828 (PMC10005692; doi:10.3390/nano13050828)
Supplement: Supplementary file 1 [file nanomaterials-13-00828-s001.zip › nanomaterials-2167389-supplementary.pdf]

nanomaterials

Supplementary Materials

Silver Anchored Polyaniline@Molybdenum Disulfide Nanocomposite (Ag/Pani@MoS_2) for Highly Efficient Ammonia and Methanol Sensing under Ambient Conditions: A Mechanistic Approach

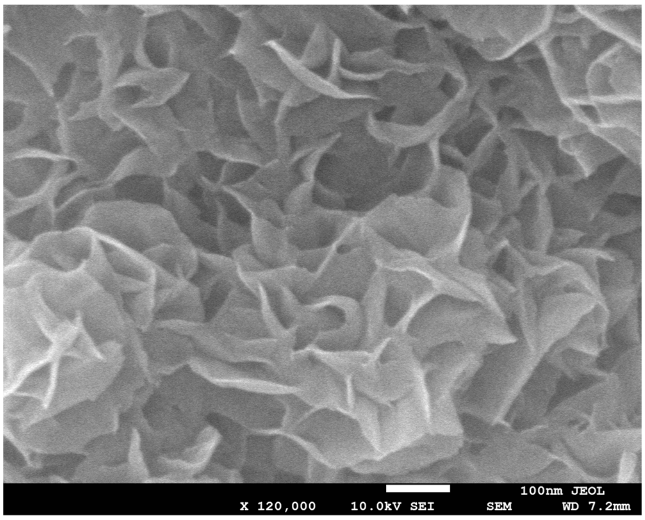

Figure. S1 SEM of MoS_2 nanosheets.

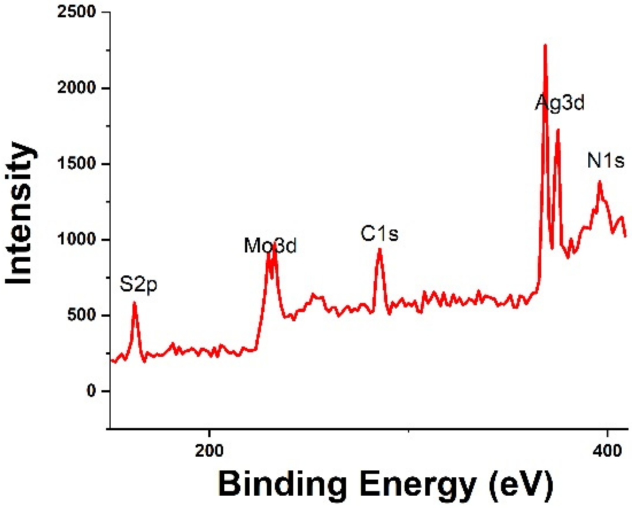

Figure. S2 XPS survey scan of *p*TSA/Ag-Pani@MoS_2

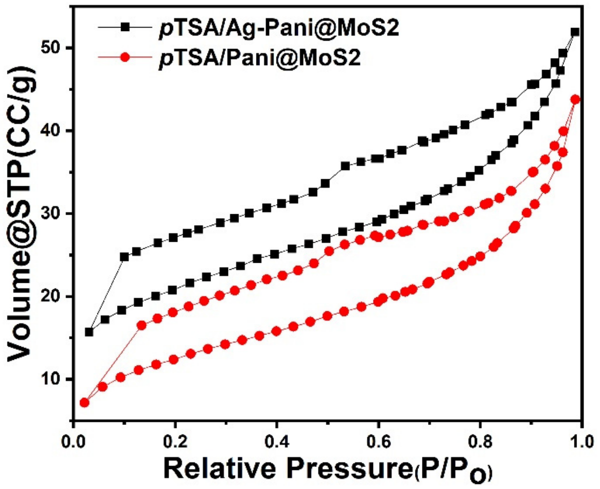

Figure. S3 absorption/desorption isotherm plots of *p*TSA/Pani@MoS_2 and *p*TSA/Ag-Pani@MoS_2

Table. S1 Surface area and pore size profile of *p*TSA/Pani@MoS_2 and *p*TSA/Ag-Pani@MoS_2

**Sample**	S_{BET}	V_{micro}	V_{meso}	V_{total}	V_{avg}
*p*TSA/Ag-Pani@MoS_2	68.84	0.0557	0.0246	0.0803	3.13
*p*TSA/Pani@MoS_2	44.65	0.0398	0.0282	0.0680	5.27
